# Droplet Digital PCR Assay for *MYD88*^*L265P*^: Clinical Applications in Waldenström Macroglobulinemia

**DOI:** 10.1097/HS9.0000000000000324

**Published:** 2020-01-03

**Authors:** Mariella Lo Schirico, Martina Ferrante, Irene Dogliotti, Alberto Zamò, Bruno Ferrero, Davide Bertuzzo, Giulia Benevolo, Paola Omedè, Federica Cavallo, Marco Ladetto, Mario Boccadoro, Daniela Drandi, Simone Ferrero

**Affiliations:** 1 Department of Molecular Biotechnologies and Health sciences, Hematology Division, University of Torino, Italy; 2 Hematology and Clinical Immunology Unit, Department of Medicine, University of Padua, Padua, Italy; 3 Division of Hematology 1, AOU “Città della Salute e della Scienza di Torino”,Torino, Italy; 4 Department of Oncology, Pathology Division, University of Torino, Italy; 5 Rita Levi Montalcini’ Department of Neuroscience, Neurology Division, University of Torino, Torino, Italy; 6 Division of Hematology 2, AOU ”Città della Salute e della Scienza di Torino”, Torino, Italy; 7 Division of Hematology, AO SS Antonio e Biagio e Cesare Arrigo, Alessandria, Italy.

Waldenström Macroglobulinemia (WM) is defined by the presence of an indolent lymphoplasmacytic lymphoma (LPL) (monoclonal lymphocytes, lymphoplasmacytes and plasma cells (PC) in the bone marrow (BM)) and monoclonal IgM protein secretion.^1,2^ Therefore, a BM biopsy showing LPL infiltration is currently essential to define WM; nevertheless, sometimes the pathological diagnosis can be troublesome: actually, the prevalence of monoclonal PC might suggest a diagnosis of multiple myeloma (MM), while in other cases, when small lymphocyte infiltration is predominant, the differentiation with other lymphomas can represent a challenge, both at morphologic and at immunophenotypic exam. In addition, the BM biopsy is a rather invasive surgical procedure and might represent a diagnostic limitation, in particular when dealing with elderly or unfit patients. Moreover, both the risk of relapse and the sensitivity to novel drugs are still difficult to predict in WM. Finally, some particular IgM-associated conditions (eg, demyelinating polyneuropathy) are still not well characterized.

Recently, a novel method to detect in different tissues the *MYD88*^*L265P*^ mutation,^3,4^ a hallmark of WM, has been described: the droplet digital PCR (ddPCR) assay.^5^ In this paper clinical reports of useful applications of this sensitive and reliable tool in daily practice are described, in a question & answer form.

## Might *MYD88* be useful for non-invasive differential diagnosis of WM vs IgM-MM?

**Figure 1:**
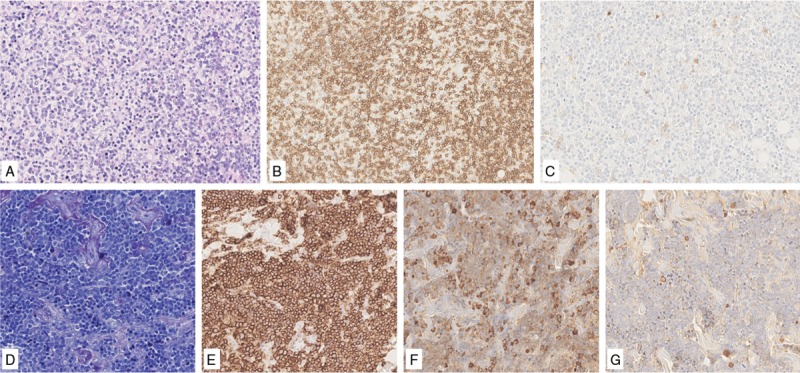
**BM biopsy showing nuomerous lymphoplasmacytic cells (A) that are CD20+ (B) and CD138- (C)**. BM biopsy showing a diffuse small cell population (D) positive for CD20 (E) and k light chain (F), negative for λ light chain (G).

MB, a 62 years-old male, presented with fatigue, dyspnea, headache and tinnitus. Blood exams revealed mild anemia (Hb 11.5 g/dl), an IgM value of 6334 mg/dl and an IgMk M-component (MC) of 3410 mg/dl, so a BM biopsy was performed (Fig. 1A–C). An excess of clonal PCs (nearly 60%) was found, at first suggesting the diagnosis of IgM-MM, while immunophenotype reported indolent B lymphoma infiltration. Interestingly, the clonal PCs did not present the chromosomal translocations typical of MM by fluorescent in situ hybridization (FISH); finally, ddPCR on BM, PB and plasma were positive for the *MYD88*^*L265P*^ mutation and a diagnosis of WM was established. The patient then started dexamethasone-rituximab-cyclophosphamide (DRC) treatment preceded by plasmapheresis, achieving partial remission (PR).

### Comment

Most of PC dyscrasias are attributable to MM, however, there are some exceptions: an IgG MC can be also attributable to rare cases of IgG-LPL or other indolent lymphomas, and similarly, some cases of IgM MC are due to aggressive IgM-MM and not to WM. In particular, because of its LPL-like pathological and phenotypical features, IgM-MM can be often mistaken for WM; however, as reported also by Treon et al,^6^ these cases are *MYD88* wild-type. So, in this setting, when laboratory findings seem discordant, or the BM biopsy is uncertain or not available, the non-invasive *MYD8*8 evaluation can support the differential diagnosis.

## Might *MYD88* be useful to refine the diagnosis of B-cell lymphoma?

AL, a 68-years-old male, presented with multiple lymphadenopathies and an IgMk MC at 680 mg/dl. The cervical lymph node biopsy was inconclusive, showing diffused small B-cell lymphoma CD20+, CD10+, BCL2+, IgD+, CD23+/−, with uncertain differential diagnosis between diffuse follicle center and marginal zone lymphoma, MZL. The BM biopsy revealed a small B-cell population with secretory differentiation and clonal IgMk lymphoplasmacytic population (20%) (Fig. 1D–G). The *MYD88*^*L265P*^ detection by ddPCR in BM and plasma finally supported the diagnosis of WM, so the patient underwent a DRC therapy, achieving complete remission.

### Comment

The differential diagnosis of small cell lymphomas with a diffuse pattern can be troublesome. Both the lymph node and the BM biopsy can be inconclusive, even after extensive flow cytometric and immunohistochemical characterization. Therefore, the availability of a sensitive and non-invasive tool, as the ddPCR *MYD88*^*L265P*^ assay, might help in choosing the most appropriate therapy.

## Might *MYD88* be used as a non-invasive marker to early identify WM relapse?

**Figure 2:**
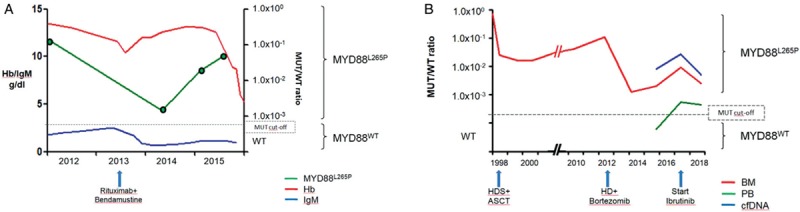
**Comparison between MYD88**^**L265P**^**, hemoglobin, and IgM values during the follow-up (A)**. MYD88^L265P^ levels in BM, PB and cell-free DNA (cfDNA) from diagnosis to the present (B).

GT, a 76-years-old female patient, was diagnosed with symptomatic WM and underwent a cyclophosphamide-vincristine-prednisone (CVP) therapy, obtaining PR. Four years later, a mild but progressive increase of IgM value was observed (2489 mg/dl), concurrently with anemia and worsening of general conditions. The patient received R-Bendamustine therapy, again achieving PR. Two years later, the patient presented with fatigue and weight loss, without worsening of blood exams (Hb 12.6 g/dl, IgM 1069 mg/dl); nevertheless, after 6 months, the appearance of pancytopenia and splenomegaly (in absence of IgM increase) suggested to repeat the BM biopsy, with a final diagnosis of progressive WM. A retrospective study of the *MYD88*^*L265P*^ mutation on PB by ddPCR showed rising values several months before the insurgence of symptoms (Fig. 2A).

### Comment

In pre-treated WM patients, the appearance of cytopenia may be due to different causes (eg, chronic blood loss anemia, myelodisplastic syndrome, acute leukemia), rather than directly related to relapsing disease.^7^ Moreover, a depletion of the secretory fraction often occurs in heavily treated patients, as well as an isolated IgM suppression may occur independently of cytotoreduction when using mTOR and BTK inhibitors,^8^ leading to discordant serological/histological results. Therefore, the mere IgM levels are not sufficiently reliable for relapse prediction. Actually, BM biopsy is essential to differentiate among these conditions, but non-invasive evaluation of the *MYD88* mutation might act as a diagnostic support.

## Might *MYD88* monitoring be used as an early response predictor to describe the activity of new treatments?

DM, a 43-years-old male, in 1997 was diagnosed with symptomatic WM and underwent high dose sequential (HDS) therapy followed by autologous stem cell transplantation. After 5 years, a slow but progressive increase of the MC was observed, leading to large lymphadenopathies and massive BM infiltration 9 years later. Therefore, a rituximab-citarabine-bortezomib therapy was started, resulting in PR. Again, after 3 years, an increase of the abdominal lymphadenopathies was observed, with anemia and rising MC: the patient started ibrutinib therapy, with complete resolution of anemia, MC reduction >50%, lymphadenopathies stability. The patient is now in good health and has been on ibrutinib treatment for 30 months.

A retrospective analysis of the *MYD88*^*L265P*^ levels in BM by ddPCR showed persistent positivity during the follow-up, with a transient, deep reduction after the bortezomib-containing therapy and a slighter but constant decrease during ibrutinib (detectable in PB and plasma, too) (Fig. 2B).

### Comment

Although WM is traditionally managed as an indolent and constantly relapsing disease, modern chemo-immunotherapies containing rituximab, bendamustine, bortezomib, as well as the new drugs carfilzomib and venetoclax^9,10,11^ resulted in major cytoreduction. Therefore, MRD analysis might provide a more accurate evaluation of the efficacy of novel treatments, rather than the simple clinical response. Actually, *MYD88*^*L265P*^ ddPCR assay can overcome the limited feasibility of the IGH-based approach,^5,12^ providing a stable molecular marker virtually to all WM patients. Moreover, the data on cell-free DNA (cfDNA) seem to nicely reflect the BM status, thus representing a non-invasive alternative for MRD detection. Nevertheless, the role of MRD in WM is not as well characterized as it is in other indolent lymphomas, so far, and its clinical impact is still under evaluation.^13^

## Might ddPCR be useful to identify MYD88 mutation in pre-treated patients?

LF, a 62-years-old male, presented with diffused lymphadenopathy and anemia (Hb 10.9 g/dl). The serum protein electrophoresis showed a MC of 1585 mg/dl (IgM value 2730 mg/dl), so lymph node and BM biopsy were performed and a diagnosis of WM was made. The *MYD88*^*L265P*^ screening by ddPCR was positive on BM. The patient underwent a DRC therapy, but at the end of treatment the CT scan revealed a SD, along with no serological response (IgM 2438 mg/dl); actually, *MYD88*^*L265P*^ resulted negative on PB, but still positive on plasma. Finally, the patient was referred to bendamustine-rituximab-bortezomib (BRB)^12^ experimental therapy.

### Comment

In patients pre-treated with rituximab, non-invasive *MYD88*^*L265P*^ evaluation in PB is not reliable because of the high rate of false negative results,^5^ likely due to the high clearance of circulating lymphoma cells. Therefore, BM or plasma analysis is advisable in pre-treated cases to identify the mutation. Actually, in paired analysis the median mutational load in PB samples is 1 log lower, compared to BM; conversely, between BM and plasma-cfDNA no statistically significant differences were reported.^5^ Moreover, a similar underestimation of *MYD88*^*L265P*^ was described in PB samples of pre-treated vs rituximab-naïve patients, both in terms of mutational detection rates (about 40% of false negatives) and of median quantitative burden (about 1 log lower).^5^

This clue is particularly relevant when *MYD88* mutational status is investigated as response predictor to targeted therapies, such as to prescribe BTK-inhibitors vs a different relapse treatment.^14^

## Might *MYD88* be useful to supplement the diagnosis of anti-mag polyneuropathy?

DF, a 54-years-old male, presented with lower limbs paresthesia. An electroneurography (ENG) showed the presence of a demyelinating polyneuropathy. The evaluation for anti-myelin-associated glycoprotein antibodies (MAG) resulted positive and the IgM value was 307 mg/dl (no MC at protein electrophoresis nor at immunofixation). A BM biopsy revealed the presence of a LPL, and the *MYD88*^*L265P*^ screening by ddPCR was positive on BM, PB and plasma. Based on the presence of LPL and a progressive anti-MAG polyneuropathy, the patient underwent 4 rituximab infusions. At the end of therapy, the IgM value was 175 mg/dl, the *MYD88* screening was negative on PB and plasma (BM biopsy was not repeated) and the ENG revealed a clear reduction of the demyelinization signs.

### Comment

Anti-MAG demyelinating polyneuropathy is a rare, disabling and still under-characterized disease that can be associated either to WM/LPL or to IgM-MGUS. The prevalence of *MYD88*^*L265P*^ mutations in anti-MAG neuropathy patients has been recently shown to be comparable to those observed in WM and MGUS control groups. Since there is actually no consensus on the optimal treatment strategy for anti-MAG neuropathies, detecting *MYD88*^*L265P*^ mutation even in non-invasive tissues might help to reveal a smoldering WM, identifying patients for which rituximab treatment may be of benefit.^15,16,17^

**Figure 3:**
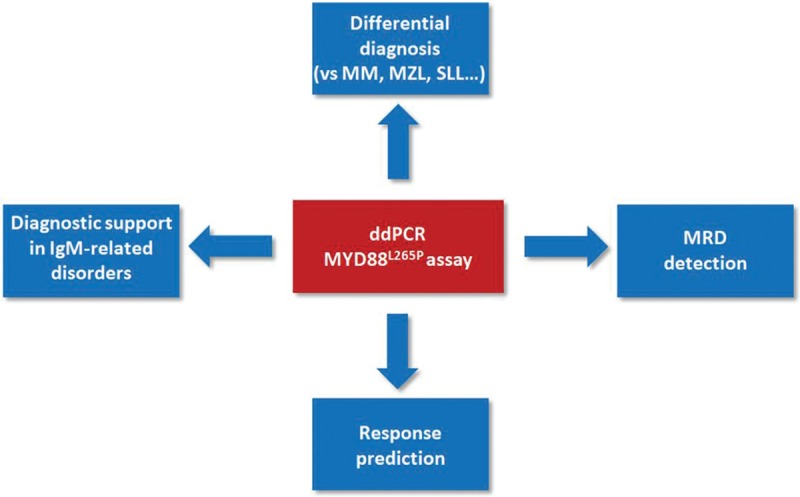
**Clinical applications of the ddPCR MYD88**^**L265P**^** assay in WM**.

In conclusion, the ddPCR *MYD88*^*L265P*^ assay might have several clinical applications (Fig. 3):

1) driving the differential diagnosis with IgM-MM and small lymphocytes, Ig-secreting disorders;
2) easy-to use molecular marker for MRD, particularly to measure the efficacy of new drugs;
3) predictive biomarker of response to ibrutinib treatment;
4) supporting the diagnosis of WM as underlying disease for rare IgM-related disorders (eg, anti-MAG polyneuropathy).

The ddPCR *MYD88*^*L265P*^ assay also presents important advantages compared to other available techniques (as qPCR or NGS): actually, it is non-invasive, cheap, fast, easily applicable to clinical routine and clinical trials, standardizable and promptly scalable to other mutations of interest (eg, *CXCR4*).

Therefore, this assay is rapidly finding its role both for mutational screening and for MRD monitoring in ongoing (BRB, *EudraCT Number: 2013-005129-22* and BIO_WM, *ID: NCT03521516*) and future (ECWM-2, *EudraCT Number: 2017-004362-95*) clinical trials.
